# Eradication of CSCs: the roadmap for curing cancer

**DOI:** 10.18632/oncoscience.516

**Published:** 2020-09-09

**Authors:** Simona Romano, Elena Cesaro, Martina Tufano, Maria Fiammetta Romano

**Affiliations:** ^1^Department of Molecular Medicine and Medical Biotechnology, University of Naples Federico II, Naples, Italy

**Keywords:** cancer stem cell, EMT, PD-L1, targeted-therapy, TAM

**Figure 1:**
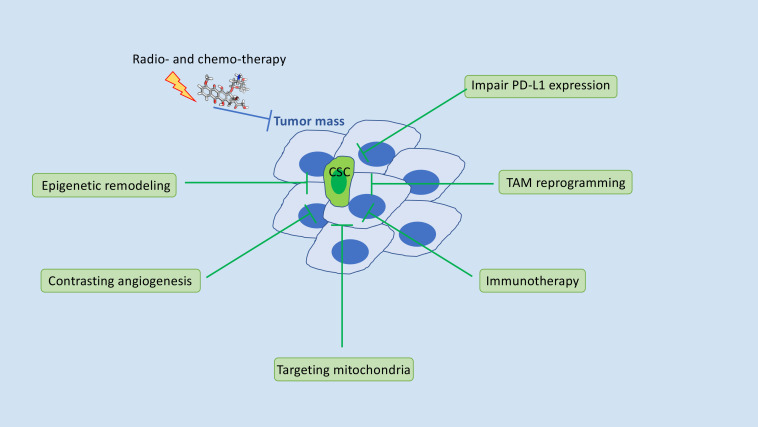
Multiple approaches for CSC disarming.

Cancer treatment failure mostly involves the insensitivity of a small, heterogeneous fraction of cancer cells within the tumor mass, endowed with extraordinary plasticity and the ability to self-renew and metastasize [1, 2]. These cells, termed cancer stem cells (CSCs) are, in general, resistant to conventional anti-cancer treatments [1, 2]. Radio- and chemotherapy can even result in the enrichment of this cell subset [1, 2]. A cancer research ambition of the last decades is to develop therapies that target the dynamic nature of CSCs and hamper the potential of non-CSCs to switch to CSC-like cells. Several strategies to target CSC-supporting pathways, that are currently under investigation in clinical trials, include impairment of mitochondria, either biogenesis and the metabolism, particularly the oxidative phosphorylation system; contrasting angiogenesis; redirect the CSC genetic program with epigenetic modulators [3]. A caveat is, however, emerging because of the intra- and inter-tumoral diversity of CSCs and the not negligible toxicity of the molecular inhibitors that undermine the success of CSC-targeted approaches [3]. CSCs have a special immunoediting capacity, a process that leads the immune system to play cooperative roles in tumorigenesis and metastasis. Recent findings show that genes active in the embryo, like Sox2, Oct4, Hippo/YAP [4], and members of the stemness-associated Wnt-signaling pathway [5] contribute to immune evasion of CSC through transcriptional activation of PD-L1 expression while disarming dendritic cells. It is worth noting that CSC-enriched subpopulations often show prominent aspects of epithelial-to-mesenchymal transition (EMT) [2]. Inherent cellular plasticity and chronic inflammatory signals associated with mesenchymal differentiation of cancer cells contribute to immune escape through multiple routes, particularly, shaping of the tumor microenvironment leads to decreased susceptibility to immune effector cells [2, 6]. In general, EMT stimulates an adaptive immune response, characterized by a significant increase in CD3+ tumor-infiltrating lymphocytes and regulatory T cells in the tumor microenvironment (TME) along with inflammation-associated cytokines, like IL-6, IL-8, and TGF-β and multiple immune regulatory molecules, including PD-L1, altogether contributing to the immunosuppressive TME [6]. The interplay between CSC/EMT and PD-L1 is bidirectional: signals generated through PD-L1 can, indeed, sustain cancer stemness and EMT genetic programs [6]. Therapies based on the so-called “immune checkpoints” have provided impressive outcomes for many tumors that are considered incurable. Particularly, PD-1/PD-L1-targeted therapy has shown enthusiastic results in clinics. The complex and dynamic interactions between tumor and TME, however, not rarely favor the immuno-escape circuits that support the intrinsic or acquired resistance to immune checkpoint blockade with antibodies. Particularly, tumor-associated macrophages (TAMs) from one side contrast immunotherapy and the other side directly communicate with CSC to promote their survival and tumorigenic potential [7]. TAMs usually express an M2 phenotype, which performs immunosuppressive and tumor-supporting functions, as the promotion of cancer cell motility, metastasis, and angiogenesis [8]. The ability of TAMs to present tumor-associated antigens is decreased as well as stimulation of the anti-tumor functions of T and NK cells [8]. Efforts to re-educate TAMs from an M2 to an M1 phenotype are being made in an attempt to make cancer more vulnerable to an immune attack. It is noticeable that treatment with antibodies blocks co-inhibitory immune receptor signaling mediated by membrane ligands/receptors. In the case of PD-L1, recent studies outline an intracellular localization of PD-L1 [9], suggesting possible functions of this molecule in intracellular compartments. Most importantly, it is increasingly emerging that PD-L1 exerts tumor-intrinsic properties, including drug resistance, cell proliferation, cell migration and invasion, and stemness phenotype [10]. Pharmacological agents that reduce PD-L1 expression were shown to exert anti-proliferative and pro-apoptotic effects in a glioma model [11]. More knowledge of genetic and molecular regulation of PD-L1 expression can lead to developing innovative tools for downmodulating PD-L1 and undermine the evil CSC/EMT/PD-L1 axis. In conclusion, the complexity and plasticity of the CSCs make it very difficult to target this subgroup of cells responsible for resistance, metastasis, and recurrence. It should be emphasized, however, that cancer research in recent years has uncovered several Achilles heels for CSCs, suggesting that tumor eradication is no longer so far. (Figure 1) resumes current approaches for CSC disarming.
